# Hand grip strength and cognitive function among elderly cancer survivors

**DOI:** 10.1371/journal.pone.0197909

**Published:** 2018-06-04

**Authors:** Lin Yang, Ai Koyanagi, Lee Smith, Liang Hu, Graham A. Colditz, Adetunji T. Toriola, Guillermo Felipe López Sánchez, Davy Vancampfort, Mark Hamer, Brendon Stubbs, Thomas Waldhör

**Affiliations:** 1 Department of Epidemiology, Center for Public Health, Medical University of Vienna, Vienna, Austria; 2 Research and Development Unit, Parc Sanitari Sant Joan de Déu, Universitat de Barcelona,Fundació Sant Joan de Déu, Dr. Antoni Pujadas, Sant Boi de Llobregat, Barcelona, Spain; 3 Instituto de Salud Carlos III, Centro de Investigación Biomédica en Red de Salud Mental, CIBERSAM, Madrid, Spain; 4 The Cambridge Centre for Sports & Exercise Sciences, Anglia Ruskin University, Cambridge, United Kingdom; 5 Department of Sport Science, Zhejiang University, Hangzhou, China; 6 Division of Public Health Sciences, Washington University School of Medicine, St. Louis, United States of America; 7 Faculty of Sports Sciences, University of Murcia, Murcia, Spain; 8 University Psychiatric Centre Catholic University Leuven, Kortenberg, Belgium; 9 School of Sport, Exercise and Health Sciences, Loughborough University, Loughborough, United Kingdom; 10 Physiotherapy Department, South London and Maudsley NHS Foundation Trust, London, United Kingdom; 11 Health Service and Population Research Department, Institute of Psychiatry, Psychology and Neuroscience, King's College London, London, United Kingdom; 12 Faculty of Health, Social Care and Education, Anglia Ruskin University, Chelmsford, United Kingdom; Ehime University Graduate School of Medicine, JAPAN

## Abstract

**Background:**

We evaluated the associations of handgrip strength and cognitive function in cancer survivors ≥ 60 years old using data from the National Health and Nutrition Examination Survey (NHANES).

**Methods:**

Data in two waves of NHANES (2011–2014) were aggregated. Handgrip strength in kilogram (kg) was defined as the maximum value achieved using either hand. Two cognitive function tests were conducted among adults 60 years and older. The Animal Fluency Test (AFT) examines categorical verbal fluency (a component of executive function), and the Digital Symbol Substitution test (DSST) assesses processing speed, sustained attention, and working memory. Survey analysis procedures were used to account for the complex sampling design of the NHANES. Multiple linear regression models were used to estimate associations of handgrip strength with cognitive test scores, adjusting for confounders (age, gender, race/ethnicity, education, marital status, smoking status, depressive symptoms and leisure time physical activity).

**Results:**

Among 383 cancer survivors (58.5% women, mean age = 70.9 years, mean BMI = 29.3 kg/m^2^), prevalent cancer types were breast (22.9%), prostate (16.4%), colon (6.9%) and cervix (6.2%). In women, each increase in kg of handgrip strength was associated with 0.20 (95% CI: 0.08 to 0.33) higher score on AFT and 0.83 (95% CI: 0.30 to 1.35) higher score on DSST. In men, we observed an inverted U-shape association where cognitive function peaked at handgrip strength of 40–42 kg.

**Conclusions:**

Handgrip strength, a modifiable factor, appears to be associated with aspects of cognitive functions in cancer survivors. Prospective studies are needed to address their causal relationship.

## Introduction

Cancer- and cancer treatment-related cognitive impairments are prevalent among cancer survivors [1]. Recent data have shown that detectable cognitive impairments are more prevalent in cancer survivors than aged matched cancer-free controls. The prevalence of cognitive impairment among cancer survivors is approximately 30% before cancer treatments, and up to 75% during- and approximately 35% post-cancer treatments [2]. This phenomenon is worse in older cancer survivors possibly owing to lower physical and cognitive reserves compared to younger survivors [1].

The modalities of preventing and treating cancer- and cancer-treatment related cognitive impairments are lacking because its aetiology is not well understood [2]. In addition, among factors that are thought to play aetiological roles, many are non-modifiable, such as host characteristics, immune dysfunction, cancer therapy induced neural toxicity, and genetics [2]. Recently, low serum level of brain-derived neurotrophic factor (BDNF) [3] was found to be associated cognitive impairments, and genetic variations in the BDNF gene [4] were found to protect against cognitive impairments in cancer patients receiving chemotherapy.

These findings are promising as BNDF is a neurotrophin, secreted in responses to muscle contraction [5]. Furthermore, muscle strength, which measures the exertion of muscle contraction, is positively related to serum BDNF concentration [6]. Meanwhile, muscle dysfunction, characterised by impairments in muscle strength or muscle composition, is common in cancer survivors [7]. However, the association between muscle function and cognitive function in cancer survivors is unknown.

To address this gap, we evaluated for the first time the cross-sectional associations of handgrip strength with two cognitive function outcomes among cancer survivors, using data from the National Health and Nutrition Examination Survey (NHANES).

## Methods

### Study population

The National Health and Nutrition Examination Survey (NHANES) was designed to provide cross-sectional estimates on the prevalence of health, nutrition, and potential risk factors among the civilian non-institutionalized U.S. population up to 85 years of age [8]. In brief, NHANES surveys a nationally representative complex, stratified, multistage, probability clustered sample of about 5,000 participants each year in 15 counties across the country. Survey participants were asked to attend physical examination in a mobile examination center (MEC). The NHANES obtained approval from the National Center for Health Statistics Research Ethics Review Board and participants provided written consent.

We extracted and aggregated data on handgrip strength, cognitive function test scores, other characteristics and cancer diagnosis from NHANES in 2011 to 2012, and 2013 to 2014. We excluded those who were never diagnosed with cancer. All NHANES data were fully anonymized before access and analysis for this study.

### Cancer diagnosis

Participants were considered to be cancer survivors if the answered affirmatively to the question “Have you ever been told by a doctor or other health professional that you had cancer or a malignancy of any kind?” We excluded participants who had non-melanoma skin cancer.

### Cognitive functioning test

Data on two cognitive function tests, the Animal Fluency Test (AFT) and the Digital Symbol Substitution test (DSST), were extracted, assessing the most commonly reported cognitive impairments in cancer survivors: executive function (working memory), and attention and concentration (processing speed) [1]. Both tests have been widely used in large-scale screenings, epidemiological and clinical studies [9–11]. For each test, cognitive function was evaluated by a score that summarizes the total number of correct answers within a given time period. *AFT* examines categorical verbal fluency, a component of executive function. The task in *AFT* asked participants to name as many animals as possible in one minute, where the total number of named animals was summarized as the test score (age-adjusted cut-offs for cognitive impairment: 65–74 years old: 15; 75–79 years old: [14]). *DSST* is a performance module from the Wechsler Adult Intelligence Scale (WAIS III), assessing processing speed, sustained attention and working memory [12]. Task in the *DSST* provided each participant a paper form that has a key at the top containing 9 numbers paired with symbols. Then participants were asked to copy the corresponding symbols in the 133 boxes that adjoin the numbers in two minutes, where the total number of correct matches was summarized as the test score (maximum score is 133 points). The cognitive function tests were administered to participants aged 60 years and older, only.

### Handgrip strength

The handgrip strength test protocol is detailed in the NHANES Muscle Strength Procedures Manual [13]. Participants did not perform handgrip strength tests if they were unable to hold the dynamometer with both hands for reasons including: missing both arms, both hands, thumbs on both hands, or paralyzed in both hands. Other reason for not performing the test included had no time, arrived late or left early, refusal, illness, emergency or equipment failures. In brief, handgrip strength in kilogram (kg) was measured with the Takei Digital Grip Strength Dynamometer over three trials separately by 60 seconds and alternating hands. Participants were asked to squeeze the dynamometer for a practice trial using submaximal effort to determine their understandings on the procedure and the grip size adjustments. They were randomly assigned to start the test with their dominant or non-dominant hand. To complete the test, participants were asked to use one hand to squeeze the dynamometer as hard as possible, and repeat using the other hand for a total of three alternating hands. Similar to previous studies using this measure, we extracted the maximum value achieved using either hand as the summary measure [14]. Studies have retrieved consistent results irrespective of using maximum or average values [15]. We classified cancer survivors as sarcopenic based on the handgrip strength criteria (men<30 kg; women <20 kg) [16].

### Socio-demographic characteristics

Data on age, sex, race and ethnicity, education and smoking status were extracted. Based on self-reported race and ethnicity, participants were classified into one of the three racial/ethnic groups: Non-Hispanic White, Non-Hispanic Black, and Hispanic and others. Participant’s education levels were classified into four groups: less than high school, high school, some college, and college graduate or above. Marital status was summarized into two groups: live with someone (married, and living with partner), and live alone (widowed, divorced, separated, never married). Finally, we classified participants into three smoking groups: never smokers (did not smoke 100 cigarettes in life and do not smoke now), former smokers (smoked 100 cigarettes in life and do not smoke now), and current smokers (smoked 100 cigarettes in life and smoke now).

### Body mass index (BMI)

Weight and height were measured at the time of physical examination in the MEC. The measurements followed standard procedures and were carried out by trained technicians using standardized equipment. BMI was calculated as weight in kg/(height in meters)^2^, and categorized into standard BMI categories: underweight (<18.5kg/m^2^), normal weight (18.5 to <25 kg/m^2^), overweight (25 to <30 kg/m^2^), and obese (≥30.0 kg/m^2^). For analytic purposes, we combined underweight and normal weight (<25 kg/m^2^).

### Depressive symptoms

Depressive symptoms were assessed using the Patient Health Questionnaire (PHQ-9), a valid 9-item depression screener asking about the frequency of symptoms of depression over the past 2 weeks [17]. Each item was scored on a 0–3 scale. The total score of FHQ-9 ranged from 0 to 27, and were categorised as “none or minimum” (0–4), “mild” (5–9), “moderate” (10–14), “moderately severe” (15–19), and “severe” (20–27) for depression severity. For current analyses, participants who scored 10 or more were combined into one group as *clinically relevant* depression, such diagnosis has shown a sensitivity of 88% and a specificity of 88% for major depression [17, 18].

### Self-reported leisure-time physical activity (LTPA)

Participants self-reported their activity patterns using questions based on the Global Physical Activity Questionnaire [19]. Levels of LTPA were calculated as the minutes per week that participants reported participating in moderate-to-vigorous-intensity physical activity (MVPA). Participants reported the frequency, and duration of physical activity (PA) in a typical week, at vigorous and moderate intensities, respectively. We summarized the total number of minutes for PA in each intensity, where minutes spent in vigorous-intensity PA were doubled and added to the number of minutes of moderate-intensity PA to approximately equivalent the metabolic equivalent of task value [20]. Cancer survivors were classified as inactive (zero min/week MVPA), insufficiently active (<150 min/week MVPA), and sufficiently active (≥150 min/week MVPA) based on the physical activity guidelines for cancer survivors [21].

### Statistical analysis

Survey analysis procedures were used to account for the sample weights (MEC weight), stratification, and clustering of the complex sampling design to ensure nationally representative estimates. NHANES cancer survivors with complete information on handgrip strength, cognitive function and other characteristics were included in the analyses. Descriptive characteristics were analysed separately in men and women, due to the documented gender difference in muscle strength [22] and cognitive function during later-life [23]. We summarized weighted means and standard errors for continuous variables, weighted proportions for categorical variables, and provided explorative *P-*values for gender comparison.

Linear regressions were carried out to quantify associations between handgrip strength and cognitive function test scores. We tested for the interaction of handgrip strength and gender and found significant terms in linear regression models for both cognitive test scores (both *P*-values<0.03). Hence, all analyses were carried out in gender-specific models. The multivariable linear regression models were adjusted for age, race, BMI, education level, smoking status, depressive symptoms and level of LTPA. We examined the normality of residuals by kernel density estimate and standardized normal probability plots for all the linear regression models. For explorative purpose, we further adjusted for the handgrip strength squared term in quadratic regressions.

The following sensitivity analyses were conducted: 1) using handgrip strength defined as the maximal value of dominant hands; 2) using handgrip strength defined as the sum of the maximal value of both hands in the multivariable regression models, for men and women, respectively. All statistical significance was set at *p*<0.05. All statistical analyses were performed using SAS version 9.4 (SAS Institute Inc., Cary, NC, USA).

## Results

A total of 434 cancer survivors had sufficient data on two cognitive function tests. Of these, we excluded 38 (9%) participants who did not have complete data on handgrip strength. We further excluded13 (3%) participants who did not provide information on other characteristics. Our analysed sample consisted of 383 cancer survivors (mean age = 70.9 years, mean BMI = 29.3 kg/m^2^) with detailed data for analyses. Prevalent cancer types were breast (22.9%), prostate (16.4%), colon (6.9%) and cervix (6.2%). There were more female (58.5%) than male cancer survivors in our sample. We observed no statistically significant differences between men and women for most characteristics, except for women being more likely to live alone (*P-*value <0.001), more likely to be non-smoker (*P-*value = 0.02) and more likely to have mild or clinically relevant depression (*P-*value = 0.01) than men (Table 1). There were also no statistically significant associations between handgrip strength defined sarcopenia (*P*-value = 0.07) or test scores on *AFT* (*P*-value = 0.07) and *DSST* (*P*-value = 0.48). Women had significant lower handgrip strength (24.0 vs. 39.8 kg, *P*-value <0.001) compared to men.

**Table 1:** Characteristics of cancer survivors aged 60 years or older from the NHANES (2011–2014), by gender.

		Men	Women	*P-*values
	*N*	183	200	
Age (years)	Mean (s.e.)	70.9 (0.6)	70.9 (0.6)	0.99
BMI (kg/m^2^)				0.27
<18.5	%	0.0	1.8	
18.5–24.9	%	21.6	28.7	
25.0–29.9	%	37.2	28.7	
≥ 30	%	41.2	40.8	
Race				0.45
Non-Hispanic White	%	84.1	86.3	
Non-Hispanic Black	%	8.6	6.0	
Hispanic and Other	%	7.3	7.7	
Education				0.28
Less than 12th grade	%	9.8	11.0	
High School	%	19.5	24.6	
Some college	%	26.6	34.3	
College graduate or above	%	44.1	29.1	
Marital status				< .001
Live with someone	%	83.0	53.9	
Live alone	%	17.0	46.1	
Smoking				0.02
Never smoker	%	34.6	53.1	
Former smoker	%	55.5	36.0	
Current smoker	%	9.9	10.9	
Depressive symptoms				0.01
None or minimum	%	88.0	70.1	
Mild	%	8.2	23.8	
Clinically relevant	%	3.8	6.1	
Leisure time physical activity (LTPA)				0.11
Inactive	%	49.3	63.5	
Insufficiently Active	%	19.2	12.1	
Sufficiently Active	%	31.5	24.4	
Handgrip strength defined sarcopenia	%	10.9	20.4	0.07
The Animal Fluency test	Mean (s.e.)	18.7 (0.6)	17.5 (0.4)	0.07
The Digital Symbol Substitution test	Mean (s.e.)	50.7 (1.6)	52.5 (1.6)	0.48

### Associations between handgrip strength and cognitive function

**Fig 1:**
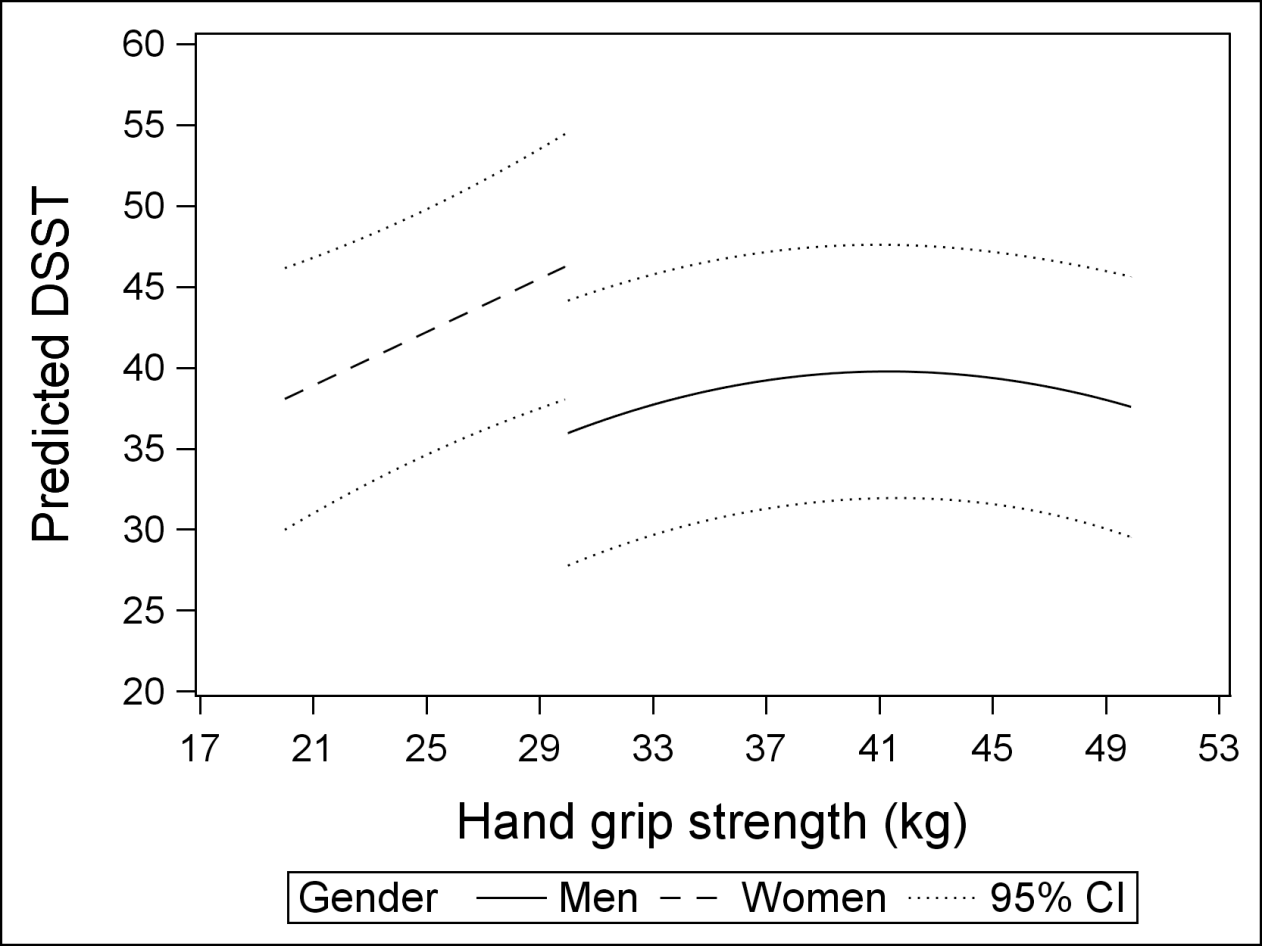
Gender-specific associations between handgrip strength and the digital symbol substitution test score among cancer survivors aged 60 years or older from the NHANES (2011–2014).

Table 2 summarizes both the unadjusted and adjusted associations between handgrip strength and cognitive function test scores. For men, high handgrip strength was associated with higher scores in both *AFT* and *DSST* in un-adjusted linear regression models. However, in multivariable-adjusted linear regression models, associations were attenuated to null. The explorative quadratic regression indicated an inverted U-shape relationship between handgrip strength and *DSST* score in men. Higher handgrip strength was associated with better *DSST* score among men, which peaked at handgrip strength of 40–42 kg. Following handgrip strength beyond 42kg, the DSST score appeared to decline. For women, those with higher handgrip strength performed better in cognitive tests in the unadjusted linear regression model, and these findings were maintained in multivariable analyses. Among women, each increase kg of handgrip strength was associated with 0.20 (95% CI: 0.08 to 0.32) and 0.83 (95% CI: 0.30 to 1.35) higher scores in the *AFT* and *DSST*, respectively. Using 10^th^ and 90^th^ percentiles of gender specific handgrip strength, Fig 1 depicts the fitted linear line and quadratic curve between handgrip strength and DSST score in men and women, respectively. We conducted further analyses restricted to breast cancer survivors (n = 93), because it was the most prevalent cancer type in our sample. We observed similar associations as we did in the analyses in all women cancer survivors, yet with slightly larger beta-coefficients. Among breast cancer survivors, each increase kg in handgrip strength was associated with 0.27 (95% CI: 0.00 to 0.54) higher score in *AFT*, and 1.03 (95% CI: 0.55 to 1.51) higher score in *DSST*.

**Table 2:** Associations between handgrip strength and cognitive function from unadjusted and multivariable linear and quadratic regression models among cancer survivors aged 60 years or older from the NHANES (2011–2014).

	Unadjusted Beta-coefficient (95% CI)	*P-* values	Adjusted^a^ Beta-coefficient (95% CI)	*P-* values
	**The Animal Fluency Test**			
Men (*n* = 183)	0.25 (0.09 to 0.41)	0.002	0.14 (-0.02 to 0.31)	0.083
Women (*n* = 200)	0.34 (0.21 to 0.46)	< .001	0.20 (0.08 to 0.32)	0.002
Breast cancer survivors (*n* = 93)	0.67 (0.14 to 0.68)	0.004	0.27 (0.00 to 0.54)	0.054
	**The Digital Symbol Substitution Test**			
Men (*n* = 183) ^b^	0.63 (0.29 to 0.96)	0.001	2.46 (1.44 to 3.47)	< .001
			-0.03 (-0.04 to -0.02) ^c^	< .001
Women (*n* = 200)	1.33 (0.91 to 1.74)	< .001	0.83 (0.30 to 1.35)	0.003
Breast cancer survivors (*n* = 93)	1.59 (0.98 to 2.20)	< .001	1.03 (0.55 to 1.51)	< .001

^a^Adjusted for age, BMI, race and ethnicity, education, marital status, smoking status, depressive symptoms and level of leisure-time physical activity.

^b^Explorative analyses fitted a quadratic regression model for the association of handgrip strength and the Digital Symbol Substitution test score in men.

^c^Handgrip strength squared.

Sensitivity analyses returned similar results. In women, using the maximal value of dominant hands, each increase kg of handgrip strength was associated with 0.17 (95% CI: 0.05 to 0.28) and 0.77 (95% CI: 0.27 to 1.26) higher scores in *AFT* and *DSST*, respectively. While using the sum of the maximal value of both hands, each increase kg of handgrip strength was associated with 0.08 (95% CI: 0.01 to 0.14) and 0.40 (95% CI: 0.09 to 0.71) higher scores in *AFT* and *DSST*, respectively. Similar quadratic regression terms were seen in men with inverted U-shape association when using maximal of dominant hands, whereas no associations were seen using sum of the maximal value of both hands (data not shown).

## Discussion

In a US nationally representative sample of 383 (weighted size = 7,489,109) cancer survivors, we investigated the associations of handgrip strength and cognitive function. Our findings suggest that stronger handgrip strength was associated with better performances on cognitive tests among women cancer survivors age 60 years and older, particularly breast cancer survivors. In men, we observed an inverted u-shape association such that higher handgrip strength was associated with better cognitive function among those having a lower level of handgrip strength, and this association reversed beyond handgrip strength of 40–42 kg. These findings suggest a role of muscle function in cancer and cancer treatment related cognitive impairments, though the drivers of the inverted u-shape association in men are not clear.

To the best of our knowledge, this is the first study to investigate the association of handgrip strength with cognitive test scores in cancer survivors. Handgrip strength is a non-invasive measure of physical health that has been widely used in research and clinical settings [24, 25]. Prior studies have established an association between handgrip strength and cognition in aging cohort studies [26, 27]. Therefore, handgrip strength has been suggested as a useful tool in geriatric practice in monitoring cognitive function decline [28]. It is worth mentioning that, although not all, most aging studies used the mental state examination as an outcome measure, which indicates global cognition [26, 29]. Nevertheless, cognition is a multi-domain concept that describes the mental process of acquiring knowledge, including aspects such as awareness, perception, reasoning and judgement [30, 31]. Among cancer survivors, the most commonly reported cognitive impairments fall in the following domains: executive function (working memory), and attention and concentration (processing speed) [1]. Therefore, it is of utmost importance for cognition measures in cancer survivor population to be domain-sensitive to cognitive impairments related to cancer and its treatment.

Previously often referred as “chemo brain”, cancer survivors experience neuropsychological difficulties after chemotherapy [32]. Prior aetiological studies have focused on cancer therapy related risk factors; such that systematic therapy (chemotherapy, radiation, and hormone therapy) agents are thought to accelerate the neurodegeneration and induce hippocampus dysfunction [33]. However, cognitive impairments are not exclusively presented among those who underwent systemic therapy. The prevalence of detectable cognitive impairments was about 30% among cancer survivors before starting treatments, higher than their aged matched cancer-free controls [2], suggesting risk factors other than cancer therapy alone.

A few pathways have been proposed to underlie the complex aetiology of cancer- and cancer treatment related cognitive impairments, such astumor biology and poor DNA repair mechanisms, generally being non-modifiable [2]. Recently work has shown promising results by demonstrating associations of inflammation, cytokines and growth factors with cognitive impairments in cancer survivors [34]. One of such markers, lower serum level of BDNF has been shown to be associated with cognitive impairments in a sample of 59 cancer patients receiving chemotherapy for metastatic disease [3]. Furthermore, findings from a longitudinal study of 145 early-stage breast cancer patients suggested that carriers of BDNF Met allele could protect cancer patients against post chemotherapy cognitive impairment [4]. BDNF is a member of the nerve growth factor family of peptides and regulates neuronal development and plasticity [35]. This finding is important because BNDF could be produced by brain and skeletal muscle cell in response to muscle contraction, namely exercise [5]. There have been pilot studies investigating the impact of exercise on cognitive functions among cancer survivors [36]. Only one study included pre-treatment data, and none considered muscle function [36]. Such design may overlook the key role of muscle dysfunction in cognitive function because weakened muscle strength and muscle mass loss is common in cancer survivors, often before treatment begins [7]. Up to 20–30% weakened muscle strength has been reported in survivors of colorectal cancer [37], prostate cancer [38], and breast cancer [39] and linked with worsened survival [40], surgery complications [40], depression and fatigue [7]. Muscle strength is positively correlated with serum BDNF concentration [6]. Our findings point to a potential critical role of muscle dysfunction in cancer- and cancer treatment related cognitive impairments. In our sample, the prevalence of handgrip strength defined sarcopenia was 10.9% in men (<30kg) and 20.4% in women (<20kg). This discrepancy in prevalence, although not significant (*P-* value = 0.07), might contribute to the different patterns of association we observed in men and women. There is no existing evidence to explain the inverted u-shape we have observed in men. Longitudinal research is needed to explore these gender-related differences in detail.

There are a number of limitations to this study. First, the cross-sectional nature makes it impossible to determine a causal association. It is possible that cancer survivors with better cognitive function were more independent, thus they maintained better physical function, than those who were cognitively impaired. Thus, the better handgrip strength might be an indicator of better cognitive function. Second, we were not able to conduct analyses stratified by time since diagnosis because of the limited number of individual cancers. Third, self-reported physical activity did not include information on types of activities performed. It is plausible that different associations exist between activity type and handgrip, likely owning to the primary muscles engaged in specific activities. Fourth, direct comparisons between the present study and previous literature in non-cancer survivors cannot be made owing to heterogeneity in outcome measures used. Future research needs to be carried out to compare changes in grip strength between cancer survivors and non-cancer survivors. Finally, the NHANES study does not query participants’ information on cancer treatment modalities and doses, which might be associated with muscle dysfunction and cognitive function decline [7, 32]. This should be explored in further studies.

## Conclusion

To the best of our knowledge, this is the first study to demonstrate that handgrip strength is associated with some aspects of cognitive function in cancer survivors. Prospective studies are needed to address causal inference. Moreover, future studies should incorporate pre-treatment information on muscle function (muscle strength and body composition), relevant biomarkers, and measures on cognition domains that are specific to cancer and its treatment. Ideally, such studies should be conducted in patients with similar tumor biology and treatment modality to elucidate the impact of muscle function, a potential modifiable factor in reducing cancer- and cancer treatment-related cognitive impairments.

## References

[1] Ahles TA, Root JC, Ryan EL. Cancer- and cancer treatment-associated cognitive change: an update on the state of the science. Journal of clinical oncology: official journal of the American Society of Clinical Oncology. 2012;30(30):3675–86. 23008308 10.1200/JCO.2012.43.0116 PMC3675678

[2] Janelsins MC, Kesler SR, Ahles TA, Morrow GR. Prevalence, mechanisms, and management of cancer-related cognitive impairment. International review of psychiatry (Abingdon, England). 2014;26(1):102–13. 10.3109/09540261.2013.864260 PMC4084673 24716504

[3] Jehn CF, Becker B, Flath B, Nogai H, Vuong L, Schmid P, et al Neurocognitive function, brain-derived neurotrophic factor (BDNF) and IL-6 levels in cancer patients with depression. Journal of neuroimmunology. 2015;287:88–92. doi: 10.1016/j.jneuroim.2015.08.012 26439967 10.1016/j.jneuroim.2015.08.012

[4] Ng T, Teo SM, Yeo HL, Shwe M, Gan YX, Cheung YT, et al Brain-derived neurotrophic factor genetic polymorphism (rs6265) is protective against chemotherapy-associated cognitive impairment in patients with early-stage breast cancer. Neuro-oncology. 2016;18(2):244–51. doi: 10.1093/neuonc/nov162 26289590 10.1093/neuonc/nov162 PMC4724179

[5] Pedersen BK. Muscle as a secretory organ. Comprehensive Physiology. 2013;3(3):1337–62. doi: 10.1002/cphy.c120033 23897689 10.1002/cphy.c120033

[6] Tsai SW, Chan YC, Liang F, Hsu CY, Lee IT. Brain-derived neurotrophic factor correlated with muscle strength in subjects undergoing stationary bicycle exercise training. Journal of diabetes and its complications. 2015;29(3):367–71. doi: 10.1016/j.jdiacomp.2015.01.014 25682570 10.1016/j.jdiacomp.2015.01.014

[7] Christensen JF, Jones LW, Andersen JL, Daugaard G, Rorth M, Hojman P. Muscle dysfunction in cancer patients. Annals of oncology: official journal of the European Society for Medical Oncology. 2014;25(5):947–58. 24401927 10.1093/annonc/mdt551

[8] Centers for Disesae Control and Prevention. National Health and Nutrition Examination Survey. http://www.cdc.gov/nchs/nhanes.htm. Accessed June 21, 2016.

[9] Tuokko H, Griffith LE, Simard M, Taler V. Cognitive measures in the Canadian Longitudinal Study on Aging. The Clinical neuropsychologist. 2017;31(1):233–50. doi: 10.1080/13854046.2016.1254279 27830627 10.1080/13854046.2016.1254279

[10] Plassman BL, Langa KM, Fisher GG, Heeringa SG, Weir DR, Ofstedal MB, et al Prevalence of dementia in the United States: the aging, demographics, and memory study. Neuroepidemiology. 2007;29(1–2):125–32. doi: 10.1159/000109998 17975326 10.1159/000109998 PMC2705925

[11] Proust-Lima C, Amieva H, Dartigues JF, Jacqmin-Gadda H. Sensitivity of four psychometric tests to measure cognitive changes in brain aging-population-based studies. American journal of epidemiology. 2007;165(3):344–50. doi: 10.1093/aje/kwk017 17105962 10.1093/aje/kwk017 PMC2244646

[12] Chen SP, Bhattacharya J, Pershing S. Association of Vision Loss With Cognition in Older Adults. JAMA ophthalmology. 2017;135(9):963–70. doi: 10.1001/jamaophthalmol.2017.2838 28817745 10.1001/jamaophthalmol.2017.2838 PMC5710542

[13] Centers for Disesae Control and Prevention. National Health and Nutrition Examination Survey (NHANES) Muscle Strength Procedures Manual. 2011.

[14] Steiber N. Strong or Weak Handgrip? Normative Reference Values for the German Population across the Life Course Stratified by Sex, Age, and Body Height. PloS one. 2016;11(10):e0163917 doi: 10.1371/journal.pone.0163917 27701433 10.1371/journal.pone.0163917 PMC5049850

[15] Haidar SG, Kumar D, Bassi RS, Deshmukh SC. Average versus maximum grip strength: which is more consistent? Journal of hand surgery (Edinburgh, Scotland). 2004;29(1):82–4. 10.1016/j.jhsb.2003.09.012 14734079

[16] Cruz-Jentoft AJ, Baeyens JP, Bauer JM, Boirie Y, Cederholm T, Landi F, et al Sarcopenia: European consensus on definition and diagnosis: Report of the European Working Group on Sarcopenia in Older People. Age and ageing. 2010;39(4):412–23. doi: 10.1093/ageing/afq034 20392703 10.1093/ageing/afq034 PMC2886201

[17] Kroenke K, Spitzer RL, Williams JB. The PHQ-9: validity of a brief depression severity measure. Journal of general internal medicine. 2001;16(9):606–13. doi: 10.1046/j.1525-1497.2001.016009606.x 11556941 10.1046/j.1525-1497.2001.016009606.x PMC1495268

[18] Manea L, Gilbody S, McMillan D. Optimal cut-off score for diagnosing depression with the Patient Health Questionnaire (PHQ-9): a meta-analysis. CMAJ: Canadian Medical Association journal = journal de l'Association medicale canadienne. 2012;184(3):E191–6. doi: 10.1503/cmaj.110829 22184363 10.1503/cmaj.110829 PMC3281183

[19] Hallal PC, Andersen LB, Bull FC, Guthold R, Haskell W, Ekelund U. Global physical activity levels: surveillance progress, pitfalls, and prospects. Lancet (London, England). 2012;380(9838):247–57. 10.1016/S0140-6736(12)60646-1 22818937

[20] Zhao G, Li C, Ford ES, Fulton JE, Carlson SA, Okoro CA, et al Leisure-time aerobic physical activity, muscle-strengthening activity and mortality risks among US adults: the NHANES linked mortality study. British journal of sports medicine. 2014;48(3):244–9. doi: 10.1136/bjsports-2013-092731 24096895 10.1136/bjsports-2013-092731 PMC10938340

[21] Rock CL, Emond JA, Flatt SW, Heath DD, Karanja N, Pakiz B, et al Weight loss is associated with increased serum 25-hydroxyvitamin D in overweight or obese women. Obesity (Silver Spring, Md). 2012;20(11):2296–301. 10.1038/oby.2012.57 PMC3849029 22402737

[22] Perna FM, Coa K, Troiano RP, Lawman HG, Wang CY, Li Y, et al Muscular Grip Strength Estimates of the U.S. Population from the National Health and Nutrition Examination Survey 2011–2012. Journal of strength and conditioning research. 2016;30(3):867–74. doi: 10.1519/JSC.0000000000001104 26196662 10.1519/JSC.0000000000001104 PMC7197498

[23] Bonsang E, Skirbekk V, Staudinger UM. As You Sow, So Shall You Reap: Gender-Role Attitudes and Late-Life Cognition. Psychological science. 2017;28(9):1201–13. doi: 10.1177/0956797617708634 28737096 10.1177/0956797617708634

[24] Roberts HC, Denison HJ, Martin HJ, Patel HP, Syddall H, Cooper C, et al A review of the measurement of grip strength in clinical and epidemiological studies: towards a standardised approach. Age and ageing. 2011;40(4):423–9. doi: 10.1093/ageing/afr051 21624928 10.1093/ageing/afr051

[25] Veronese N, Stubbs B, Fontana L, Trevisan C, Bolzetta F, Rui M, et al A Comparison of Objective Physical Performance Tests and Future Mortality in the Elderly People. The journals of gerontology Series A, Biological sciences and medical sciences. 2017;72(3):362–8. doi: 10.1093/gerona/glw139 27470299 10.1093/gerona/glw139

[26] Clouston SA, Brewster P, Kuh D, Richards M, Cooper R, Hardy R, et al The dynamic relationship between physical function and cognition in longitudinal aging cohorts. Epidemiologic reviews. 2013;35:33–50. doi: 10.1093/epirev/mxs004 23349427 10.1093/epirev/mxs004 PMC3578448

[27] Veronese N, Stubbs B, Trevisan C, Bolzetta F, De Rui M, Solmi M, et al What physical performance measures predict incident cognitive decline among intact older adults? A 4.4year follow up study. Experimental gerontology. 2016;81:110–8. doi: 10.1016/j.exger.2016.05.008 27235850 10.1016/j.exger.2016.05.008

[28] Fritz NE, McCarthy CJ, Adamo DE. Handgrip strength as a means of monitoring progression of cognitive decline—A scoping review. Ageing research reviews. 2017;35:112–23. doi: 10.1016/j.arr.2017.01.004 28189666 10.1016/j.arr.2017.01.004

[29] Jang JY, Kim J. Association between handgrip strength and cognitive impairment in elderly Koreans: a population-based cross-sectional study. Journal of physical therapy science. 2015;27(12):3911–5. doi: 10.1589/jpts.27.3911 26834379 10.1589/jpts.27.3911 PMC4713818

[30] Brandimonte MA, Bruno N, Callina S. Cognition In: Pawlik P, d'Ydewalle G, editors. Psychological Concepts: An International Historical Perspective. Hovem UK: Psychology Press; 2006.

[31] McKhann GM, Knopman DS, Chertkow H, Hyman BT, Jack CR Jr., Kawas CH, et al The diagnosis of dementia due to Alzheimer's disease: recommendations from the National Institute on Aging-Alzheimer's Association workgroups on diagnostic guidelines for Alzheimer's disease. Alzheimer's & dementia: the journal of the Alzheimer's Association. 2011;7(3):263–9. 10.1016/j.jalz.2011.03.005 PMC3312024 21514250

[32] Moore HC. An overview of chemotherapy-related cognitive dysfunction, or 'chemobrain'. Oncology (Williston Park, NY). 2014;28(9):797–804. 25224480

[33] Janelsins MC, Kohli S, Mohile SG, Usuki K, Ahles TA, Morrow GR. An update on cancer- and chemotherapy-related cognitive dysfunction: current status. Seminars in oncology. 2011;38(3):431–8. doi: 10.1053/j.seminoncol.2011.03.014 21600374 10.1053/j.seminoncol.2011.03.014 PMC3120018

[34] Castel H, Denouel A, Lange M, Tonon MC, Dubois M, Joly F. Biomarkers Associated with Cognitive Impairment in Treated Cancer Patients: Potential Predisposition and Risk Factors. Frontiers in pharmacology. 2017;8:138 doi: 10.3389/fphar.2017.00138 28377717 10.3389/fphar.2017.00138 PMC5359273

[35] Leibrock J, Lottspeich F, Hohn A, Hofer M, Hengerer B, Masiakowski P, et al Molecular cloning and expression of brain-derived neurotrophic factor. Nature. 1989;341(6238):149–52. doi: 10.1038/341149a0 2779653 10.1038/341149a0

[36] Zimmer P, Baumann FT, Oberste M, Wright P, Garthe A, Schenk A, et al Effects of Exercise Interventions and Physical Activity Behavior on Cancer Related Cognitive Impairments: A Systematic Review. BioMed research international. 2016;2016:1820954 doi: 10.1155/2016/1820954 27144158 10.1155/2016/1820954 PMC4842032

[37] Burden ST, Hill J, Shaffer JL, Todd C. Nutritional status of preoperative colorectal cancer patients. Journal of human nutrition and dietetics: the official journal of the British Dietetic Association. 2010;23(4):402–7. 20487172 10.1111/j.1365-277X.2010.01070.x

[38] Soyupek F, Soyupek S, Perk H, Ozorak A. Androgen deprivation therapy for prostate cancer: effects on hand function. Urologic oncology. 2008;26(2):141–6. doi: 10.1016/j.urolonc.2006.12.014 18312932 10.1016/j.urolonc.2006.12.014

[39] Harrington S, Padua D, Battaglini C, Michener LA, Giuliani C, Myers J, et al Comparison of shoulder flexibility, strength, and function between breast cancer survivors and healthy participants. Journal of cancer survivorship: research and practice. 2011;5(2):167–74. 21225372 10.1007/s11764-010-0168-0

[40] Chen CH, Ho C, Huang YZ, Hung TT. Hand-grip strength is a simple and effective outcome predictor in esophageal cancer following esophagectomy with reconstruction: a prospective study. Journal of cardiothoracic surgery. 2011;6:98 doi: 10.1186/1749-8090-6-98 21843340 10.1186/1749-8090-6-98 PMC3170319

